# Ultra-processed food exposure and cognitive outcomes: a systematic review of observational studies

**DOI:** 10.1136/bmjnph-2025-001325

**Published:** 2026-05-04

**Authors:** Megan Smith, Pippa Watson, John Gallacher, Sarah Bauermeister

**Affiliations:** Department of Psychiatry, University of Oxford, Oxford, UK

## Abstract

Ultra-processed food (UPF) intake has been associated with negative health outcomes. Research investigating UPF intake and cognitive health outcomes has begun. The aim of this review is to summarise the existing evidence of associations between exposure to UPFs, as defined by the NOVA food classification system, and cognitive health outcomes. We conducted a systematic search across multiple databases for relevant studies up to October 2024. The Newcastle-Ottawa Scale was used to assess the quality of included studies. A narrative approach was used to summarise and integrate results across studies. 383 articles were screened and five met the inclusion criteria. The association between UPF intake and four different cognitive outcomes (dementia risk, cognitive impairment risk, cognitive performance and cognitive change trajectories) was explored. Three out of the five studies found a significant negative main effect of consuming UPF on the outcome of interest. All studies identified adverse associations of consumption; for some studies, these negative associations were isolated to a subgroup of the population or a subgroup of UPF type. Conclusions should be drawn with caution due to the limited number of studies available examining UPF intake according to NOVA and its association with cognitive outcomes, as well as the variability in cognitive measures assessed. Due to the novelty of this research area, more studies are required to help elucidate whether, and how, UPF may affect cognitive health. Additionally, future analyses should incorporate a measure of overall diet quality to aid in determining whether the effect of UPF is independent of dietary pattern or influenced by it.

PROSPERO registration number CRD42024600338.

## Introduction

Currently, more than 55 million people are living with dementia worldwide.^1^ The social, economic and healthcare burden of dementia is vast, due to those living with the condition often experiencing a reduction in independence.^2^ Although clinically distinct, dementia and cognitive function exist on a continuum whereby dementia represents an advanced stage of cognitive decline that is often preceded by measurable impairments in cognition.^3^ Prevention strategies and risk reduction play an integral role in managing the incidence of dementia and cognitive decline. These strategies assist by slowing or averting the neurodegenerative processes that lead to cognitive decline and dementia. Many risk factors are lifestyle or ‘modifiable’ factors, such as diabetes, cholesterol and hypertension.^4^ Several of these modifiable risk factors can be adjusted by diet, meaning a person’s risk of developing cognitive decline or dementia may be modulated through factors related to dietary intake. For instance, an unhealthy diet can contribute to the development of type 2 diabetes (a risk factor for dementia) while a balanced, nutrient-dense diet can prevent diabetes onset^5^ and thus mitigate the associated dementia risk.

While multiple definitions of ultra-processed foods (UPFs) exist, the most widely used is the NOVA classification system.^6^ This framework classifies all food items according to their degree and purpose of processing. According to this classification, UPFs are manufactured items containing minimal whole foods, alongside numerous substances that are of little or no culinary use (eg, high-fructose corn syrup and modified starches), which are added to enhance the flavour, texture and/or the shelf-life of the product. UPFs are now the main - or major - source of dietary energy in many high-income countries, including the UK and USA.^7 8^ Consumption patterns in low- to middle-income countries have begun demonstrating a shift away from traditional diets and towards UPFs.^9^

Large-scale epidemiological studies highlight an increased risk of nearly all major non-communicable diseases associated with high UPF intakes. Illustratively, high UPF consumption has been associated with an increased risk of all-cause mortality,^10^ cardiovascular disorders (CVDs),^11^ type 2 diabetes,^12^ cancers^13^ and obesity^7^ risk. A recent umbrella review found that greater UPF exposure was associated with a higher risk of adverse health outcomes.^14^ Discussions regarding public health strategies and policies to limit UPF intake have consequently become salient. Systematic reviews aid the debate by consolidating evidence, identifying knowledge gaps and clarifying discrepant findings.

It is plausible that UPF intake might influence cognitive health and dementia risk as biological pathways implicated in maintaining brain health, such as inflammation and the gut microbiome, are modulated by UPF intake.^15 16^ However, investigations on the cognitive effects of consuming UPFs have been initiated with conflicting results.

Evidence on UPF and cognition is growing as the debate of UPFs contribution to adverse health outcomes has gained traction. Two systematic reviews have summarised the evidence regarding UPF intake and dementia^17^ and Alzheimer’s disease^18^ risk. Further studies have reviewed associations between individual UPF items and cognitive health outcomes, for instance, sugar-sweetened beverages and cognitive disorders.^19^ However, evidence on total UPF intake and multiple cognitive health outcomes has not been reviewed. Here, we address this gap by considering only research which has assessed the level of processing using the NOVA classification system and including cognitive functioning, cognitive decline and dementia risk as outcomes.

## Methods

We conducted and reported this review in accordance with the Preferred Reporting Items for Systematic Reviews and Meta-Analyses (PRISMA) guidelines. The review was pre-registered on PROSPERO (CRD42024600338).

### Inclusion criteria

The inclusion criteria were constructed in line with the population, exposure, comparisons, outcomes and study design reporting structure. Therefore, studies involving human adults (over 18 years of age) who were free from dementia at baseline (population) were eligible. The intake of UPFs needed to be measured and defined by the NOVA classification system (exposure). The comparison required was higher versus lower UPF exposure. Studies were required to investigate associations between intake and cognitive performance, cognitive performance trajectories, cognitive impairment or dementia risk (outcome). Epidemiological observational studies, including prospective cohort, case–control and/or cross-sectional studies (study design), were included. The full exclusion and inclusion criteria are in online supplemental table 1.

Studies which measured the intake of only one type of UPF (eg, sugar-sweetened beverages) or measured a diet high in UPF but did not specifically investigate overall UPF intake (eg, studies which explored ‘western diet patterns’, which are characteristically high in UPFs but include other non-UPF items) were not included in this review.

### Search strategy

One author (MS) conducted a systematic search across MEDLINE, PsycINFO, Embase and APA Psych Articles through OVID, and PubMed for relevant studies up to October 2024. The search terms were words relevant to UPFs, NOVA classification and cognitive outcomes (full search terms in online supplemental table 2).

The identified studies were exported to the Rayyan software. Duplicates were removed before two reviewers independently determined eligible studies. The initial selection was based on titles and abstracts, with studies deemed potentially relevant being read in full before being included or excluded. Any disagreements between reviewers were resolved by a third reviewer.

### Data extraction

One reviewer completed data extraction (MS). Extracted data included study characteristics such as publication year, country, sample size and sample characteristics (eg, % female, mean age). Data relevant to the methods, including dietary intake assessment, dementia diagnosis or cognitive performance measurement, and statistical methods used were extracted. The results obtained between assessing UPF intake and dementia risk or cognitive performance (ie, HR or OR and 95% CIs, or beta-values for association scores and 95% CIs), were extracted. Additionally, factors required to evaluate the quality of the studies were obtained (eg, follow-up duration and adjustment of confounders).

### Quality of evidence

To assess the quality of evidence, we applied the Newcastle-Ottawa scale (NOS).^20^ This tool consisted of eight items, evaluating the quality of the study over three domains (selection, comparability and outcome). The total maximum score was 9 (1 point available for all but one item, where 2 points were available). Studies obtaining scores lower than 5 points were classified as poor-quality, those with 5 or 6 points as medium quality and those with 7 or more points were considered high quality.^17 21^ Where appropriate, we used the adapted version of the NOS for the specific assessment of cross-cohort studies. Two reviewers (MS and PW) independently rated the included evidence, with any disagreement resolved by a third reviewer.

### Data synthesis

To integrate the findings of the studies we used a narrative approach. This method was chosen due to the small number of studies and the heterogeneity in outcomes investigated. Synthesis was initially assessed across all studies to examine the overarching theme of UPF and cognitive outcomes (eg, dementia risk, cognitive performance and cognitive change). Studies were then grouped according to the cognitive outcome of interest to evaluate the evidence pertinent to these outcomes.

## Results

### Study selection and characteristics

**Figure 1:**
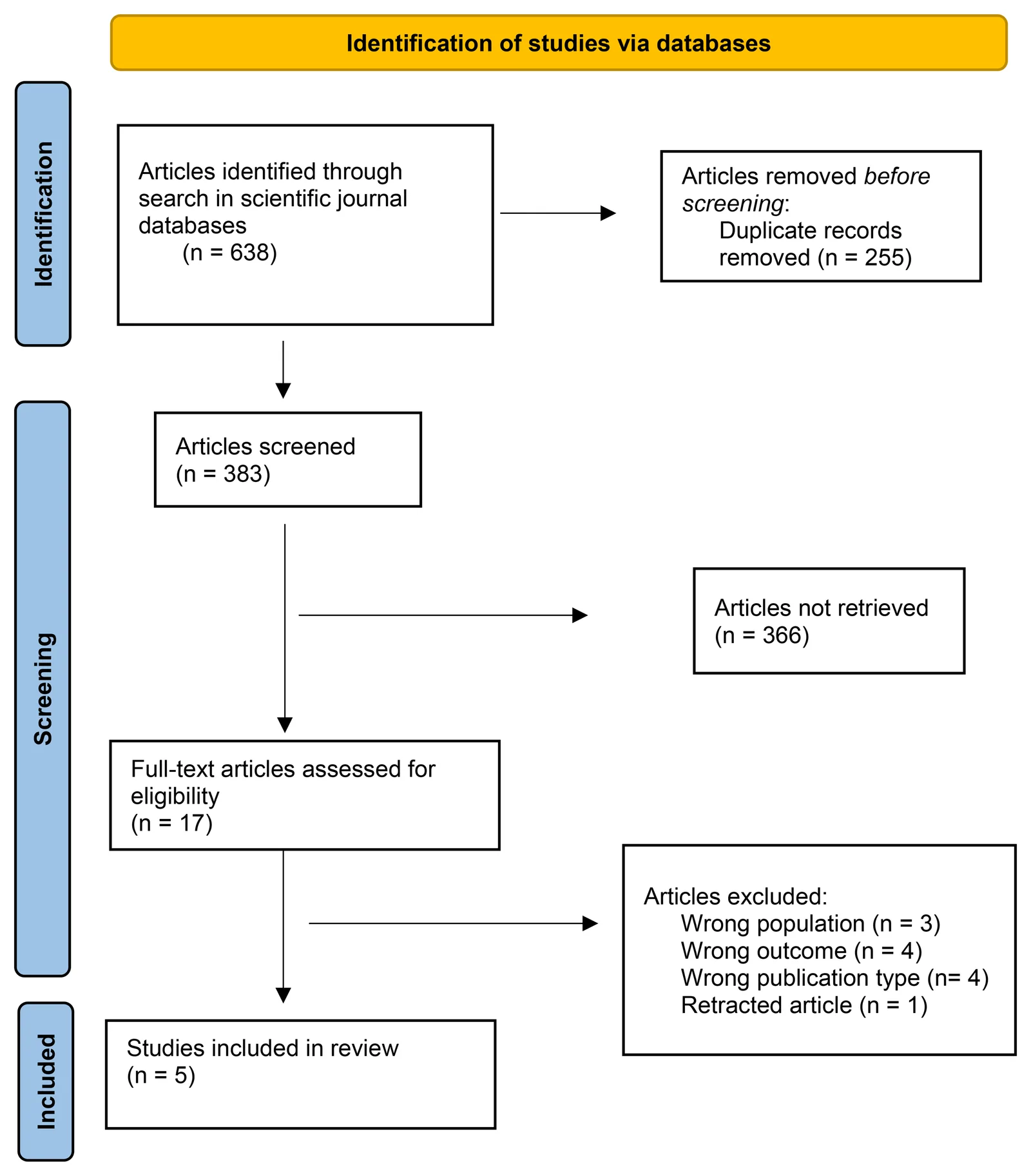
Preferred Reporting Items for Systematic Reviews and Meta-Analyses (PRISMA) flow diagram for study selection process.

A flowchart demonstrating the selection process of the studies is illustrated in figure 1. After removal of duplicates (n=255), 383 articles were screened and five met the inclusion criteria (as specified in the Inclusion criteria section).

The included studies (table 1) were conducted in the USA,^22 23^ the UK,^24^ Israel^25^ and Brazil.^26^ All studies were published recently in this review, between 2022 and 2024. One study was cross-sectional^22^ and four^23–26^ were longitudinal, with follow-ups ranging from 5.3^25^ to 10 years.^24^ Sample sizes ranged from 568^25^ to 72 083.^24^ Three studies collected dietary data via Food Frequency Questionnaires (FFQ)^23 25 26^ and two^22 24^ used 24-hour dietary recall methods. All studies included an exposure variable of the percentage of the person’s diet that is ultra-processed; in some studies^23 24^ this was expressed as a percentage of total weight (% g/day) while in others^22 25 26^ it was expressed as a percentage of total calorie intake (% kcal/day). UPF intake was modelled as a continuous exposure variable^23 24^ and as a categorical variable.^22 25 26^ Table 2 displays the outcome and statistical characteristics of included studies. The association between UPF intake and a range of outcomes was explored: one study assessed the association with dementia risk,^24^ one study evaluated the association with cognitive impairment risk,^23^ one study explored the relationship with cognitive performance^22^ and three studies estimated the association with cognitive change.^23 25 26^ Across those measuring cognitive performance (n=4), all employed a different number of tests, ranging from 3^22^ to 14.^25^ 17 unique cognitive tests were administered across the studies; of these, 10 tests were conducted in more than one study. All studies controlled for age, gender, education, physical activity and body mass index. One study^24^ did not control for any measures of diabetes and one study did not control for cardiovascular disease.^25^ Adhesion to a healthy diet was considered in three studies.^23 24 26^ Across all studies, other covariates included were income, deprivation, depression, alcohol consumption, family history of dementia, total energy intake, HbA1C, total cholesterol and blood pressure.

### Quality of evidence and risk of bias in included studies

The NOS scores varied from 6 to 8 across studies. One study was considered medium quality, with a higher risk of bias.^22^ The four other studies were rated as high quality, as NOS scores were either 7 (n=1)^25^ or 8 (n=3).^23 24 26^ See online supplemental tables 3 and 4 for NOS scoring details.

### Narrative synthesis

Three out of the five studies found a significant negative main effect of consuming UPF on the cognitive outcome of interest,^23 24 26^ while all studies highlighted a significant adverse consequence of consumption in either a subgroup of the population^22^ or a subgroup of UPF type.^25^ Overall therefore, deleterious effects of UPF consumption on multiple cognitive health outcomes were identified, but the combined results suggest possible specificity in the relationship between UPF and brain health in terms of participant characteristics (eg, health status) and/or subtype of UPF consumed. The main results of included studies are summarised in table 2.

One study^24^ assessed the relationship between UPF intake and the risk of developing dementia in 72 000 participants. A higher consumption of UPF was associated with a higher risk of dementia, Alzheimer’s disease and vascular dementia. Specifically, a 10% increase in the percentage of UPF intake was associated with a 25% increase in the risk of all-cause dementia, 14% in the risk of Alzheimer’s and a 28% increase in the risk of developing vascular dementia. To explore the effect of replacing UPF items with a less processed alternative, the authors conducted substitution analysis which highlighted how replacing 10% of UPF intake with less processed foods reduced the risk of dementia by 19% and vascular dementia by 22%. Similarly, higher UPF intake was linked to increased risk of adverse cognitive outcomes in another study^23^ identifying that a 10% increase in UPF consumption was significantly associated with a 16% higher risk of developing cognitive impairment.

A single study^22^ examined the association between UPF consumption and cognitive performance cross-sectionally. In 3632 US adults aged over 60 years, no significant association was identified between UPF intake and performance on any of the cognitive tests administered. However, in the participants without CVD or diabetes, UPF intake was inversely associated with performance on the Animal Fluency Task (a measure of language and executive function).

The relationship between cognitive change and UPF intake was investigated in three studies.^23 25 26^ Two studies found a significant association between increased UPF consumption and accelerated cognitive decline^23 26^ while one study^25^ found no relationship between total UPF intake and cognitive change. However, this study^25^ did find a relationship between specific subgroups of UPFs and cognitive change; higher intake of ultra-processed meat and oils/spreads was associated with significantly faster decline in executive functions and global cognition.

## Discussion

This systematic review provides an overview and evaluation of the evidence for the associations between UPF intake and cognitive health. The review included five studies which covered four different cognitive outcomes. The outcomes investigated were cognitive performance, cognitive decline, dementia risk and cognitive impairment risk. While not all studies highlighted a significant main effect of UPF intake on the outcome of interest, all studies identified adverse consequences of consumption in either a subgroup of the population or a subgroup of UPF type. However, conclusions should be drawn with caution due to the small number of studies available investigating NOVA classified UPF intake and cognitive outcomes, and the range of cognitive outcomes investigated across the limited available studies.

Our results largely align with the two other systematic reviews in the field of UPF and cognitive health. In a meta-analysis of UPF items, high UPF intake was associated with an increased risk of dementia,^17^ and a systematic review concluded an increased risk of Alzheimer’s disease from increased consumption of UPF items.^18^ These findings are based on reviews that included literature where total UPF intake, as defined by NOVA, was not explicitly measured. Additionally, these reviews focused on only AD and dementia diagnosis. Nevertheless, the results are consistent with our overarching cognitive outcomes review, which specifically aggregated studies measuring total UPF intake using NOVA. Also, our review reflects previous review results that have explored individual UPF items, such as sugar-sweetened beverages that concluded adverse cognitive health outcomes from consumption.^19^ Moreover, this current review echoes the broader literature of negative health consequences from increased UPF exposure.^14^

Heterogeneity in results was observed among the studies investigating UPF consumption and cognitive change, with two out of three studies highlighting the negative effects of UPF exposure on cognitive decline. The discrepant finding compared with the other studies may be attributable to the specific population included, which focused exclusively on individuals with type 2 diabetes.^25^ This explanation is supported by findings which highlighted that UPF intake was only associated with cognitive performance in individuals without pre-existing health conditions.^22^ This suggests the effect UPFs have on cognition may be modified by the overall health status of the participant, and therefore the lack of a significant main effect in one study^25^ might be attributable to participants with type 2 diabetes. Further studies are required to explore the cognitive consequences of UPF intake in other and more diverse populations.

Three of the five included studies consider diet quality (ie, healthy diet) in their investigations.^23 24 26^ Incorporating diet quality into analyses is imperative to aid the discussion of whether UPF intake confers independent adverse effects on health, or whether it is diet quality that mediates any relationship between UPFs and health outcomes. As UPFs often lack nutritional value and their intake is inversely associated with fruits, vegetables and legumes,^27^ it may simply be that UPFs are related to deleterious outcomes because they are associated with an overall unhealthy diet. The adverse effects of UPF exposure on cognitive outcomes remained while controlling for adherence to a healthy diet in two out of the three studies.^23 24^ The persistence of associations implies UPFs independently impact cognitive outcomes and therefore, it may be the nature and extent of processing that is having negative consequences. Opposingly, the negative effects of UPF intake on cognitive decline were isolated to only those who were consuming an unhealthy diet, with those who ate a healthy diet not experiencing the detrimental cognitive outcomes from UPF intake in another study,^26^ suggesting that diet quality modifies the association between UPF and brain health. Future studies could incorporate a measure of diet quality or pattern in the analysis to help explore whether the effect of consuming UPF on cognitive outcomes is independent - or not - from the quality of the diet.

The mechanisms by which UPF consumption might lead to negative health consequences, and specifically poor cognitive health, remain to be fully understood. UPF intake may influence cognitive health directly or indirectly. Direct mechanisms include a lack of nutrients supplied by a high UPF diet^27^ directly impacting brain structure and function.^28^ Indirect mechanisms include how UPFs affect systemic health which can culminate in neuropathological changes. For instance, biological pathways implicated in brain health, such as inflammation, oxidative stress and the gut microbiome, are thought to be modulated by UPF exposure.^29^ To illustrate, UPFs are linked to pro-inflammatory processes through modulating the composition of the gut microbiome,^30^ and inflammation is a pathological hallmark of cognitive decline^31^ and dementia.^32^

There was a lack of consistency in how UPF intake was modelled across studies; exposures included UPF intake as a ratio of both total energy and total food weight intake. This reflects discussions in the literature regarding the best way to capture UPF consumption. The variation in measurement may influence study findings, as the two metrics capture different dietary aspects and can classify individuals differently. Sensitivity analysis comparing results using both ratios was only conducted by one study,^23^ and importantly, this identified weaker associations between UPF intake and cognitive impairment when using the energy ratio compared with the weight ratio. However, there was no consistency across studies in one metric finding an association between UPFs and cognitive health and the other not.

Notably, the three largest studies (over 10 000 participants) found a significant main effect of UPF intake on cognitive health.^23 24 26^ The effect sizes reported in these studies were relatively small, indicating the effect of UPF intake on cognitive health might be modest. Together, these might suggest that these larger samples provided the statistical power needed to detect associations.

Although the NOVA classification system is the most widely used framework for categorising foods according to their degree of processing, it has received considerable criticism. One key limitation is the ambiguity and inconsistency involved in assigning food items to different NOVA categories, which may lead to misclassification and affect the reliability of study findings.^33^ Additionally, UPFs are often high in added fats and sugars while being low in protein, fibre and vitamins.^34^ Consequently, it remains unclear whether any observed negative associations between UPF consumption and cognitive health are driven primarily by their nutritional composition or by the processing methods themselves. Future studies could address this question by incorporating a measure of diet quality or nutrient profiles.

As evidence of the potential harmful effects of UPF consumption grows, researchers and public health organisations have been appealing for governmental action to help curb rising intake. However, the small number of studies identified in this review highlights the need for additional research to better understand the cognitive consequences of UPF consumption before findings can meaningfully inform the development of public health strategies or dietary guidelines. Additionally, this review - along with others in the broader literature of UPFs and health^14^ - noted possible heterogeneity between UPF subgroups and health outcomes. Such disparity in associations underscores the need for greater nuance in discussions surrounding UPF consumption and highlights the importance of further research to ensure that future public health messaging and dietary recommendations are clear, evidence-based and do not inadvertently contribute to confusion among the public.

Methodological differences between the studies should be noted. First, four different cognitive outcomes were explored; consequently, the ability to collate information on the specific cognitive outcomes is limited. Second, studies used different dietary assessment methods, including 24-hour recalls and FFQs. Dietary intake methods are noteworthy due to the impact on the ability to classify items as ultra-processed or not; the different tools collect varying specificity on the intake and preparation of food items and thus influence UPF classification precision. For example, 24 hours recalls allow the participant to freely record the intake along with brand information - this level of detail aids the assignment to the necessary NOVA category. Third, UPF intake was expressed as both a percentage of total calories and total weight intake. Weight-based measures are suggested to more accurately capture UPF intake than calorie-based ones due to the low-calorie nature of some UPFs^35^ and the ability to account for non-nutritional factors related to food processing.^22^ However, the variation in exposure limits the capacity to compare across studies.

The strengths of this review primarily lie with the broad outcomes included along with the specificity in only NOVA classified UPF intake. As such, this review provides a focused yet multifaceted overview of the current literature on UPF intake and cognitive outcomes. Nonetheless, our review findings should be interpreted with caution due to several limitations. Principally, only five studies were included in the review due to the novelty of the research area; the limited number of studies therefore limits the generalisability, strength and confidence in the conclusions which can be drawn. Second, substantial heterogeneity existed between the studies. Variety was observed in the cognitive outcome explored, the cognitive tests employed, the methods used to measure dietary intake, the expression of UPF intake, along with other methodological differences. Third, because all included studies were observational, this review cannot establish causal relationships, and residual confounding may persist despite statistical adjustment for covariates. Accordingly, the findings should be interpreted with caution, and we invite future well-designed randomised controlled trials to more robustly assess causality.

## Supplementary Material

This content has been supplied by the author(s). It has not been vetted by BMJ Publishing Group Limited (BMJ) and may not have been peer-reviewed. Any opinions or recommendations discussed are solely those of the author(s) and are not endorsed by BMJ. BMJ disclaims all liability and responsibility arising from any reliance placed on the content. Where the content includes any translated material, BMJ does not warrant the accuracy and reliability of the translations (including but not limited to local regulations, clinical guidelines, terminology, drug names and drug dosages), and is not responsible for any error and/or omissions arising from translation and adaptation or otherwise.

Supplementary file

## Figures and Tables

**Table 1:** Characteristics of included studies

Authors	Country (cohort)	Study design	Sample size	Follow-up duration years	Age (mean, SD)	Sex (% female)	Dietary data collection method	Level of exposure (expressed as)
Cardoso *et al*^22^	USA (NHANES)	Cross-sectional cohort	2713	N/A	69.1 (0.2)	54% female	24 hours dietary recall	Tertiles (% kcal/day)
Li *et al*^24^	UK (The UK Biobank)	Prospective cohort	72 083	Median 10	61.6 (3.94)	52.9	24 hours dietary recall	Continuous 10% increase (% g/day)
Weinstien *et al*^25^	Israel (IDCD)	Prospective cohort	568	Mean 5.3	71.3 (4.5)	40	FFQ	Quartiles (% kcal/ day)
Gomes Gonçalves *et al*^26^	Brazil (ELSA-Brazil)	Prospective cohort	10 775	Median 8	51.6 (8.9)	54.6	FFQ	Quartiles (% kcal/ day)
Bhave *et al*^23^	USA (REGARDS)	Prospective cohort	14 175	Mean 10.7	63.5 (8.6)	58	FFQ	Continuous 10% increase (% g/day)

ELSA-Brazil, Brazilian Longitudinal Study of Adult Health; FFQ, Food Frequency Questionnaire; IDCD, Israel Diabetes and Cognitive Decline; NHANES, National Health and Nutrition Examination Survey; REGARDS, Reasons for Geographic and Racial Differences in Stroke.

**Table 2:** Analysis methods, main results and conclusions of included studies

Authors	Outcome	Outcome measurement	Statistical analysis	Main results	Conclusions	Effect modifiers	Covariates
Cardoso *et al*^22^	Cognitive performance	Three cognitive tests: Consortium to Establish a Registry for Alzheimer’s Disease (CERAD), Word Learning test, Animal Fluency test and the Digit Symbol Substitution test (DSST)	Linear regression models	No significant association between UPF intake and performance on any cognitive test. Among individuals with no pre-existing diseases, those in the highest tertile of UPF consumption performed significantly worse on the Animal Fluency test (β=−1.37, 95% CI −2.72 to −0.03, p=0.046) compared with the lowest consumption tertile	UPF intake was not associated with cognitive performance in the full sample. There may be an interaction between UPF consumption and health status as higher UPF consumption was associated with worse performance on the Animal Fluency task, but only among those with no CVD or diabetes	Health status	Age, gender, ethnicity, education, poverty-income ratio, physical activity, smoking status, BMI, CVD, diabetes and depression
Li *et al*^24^	All-cause dementia	Linkage with hospital admission data and death registers	Cox proportional hazards regression models	Increasing UPF intake by 10% was associated with a 25% significant increase in the risk of all-cause dementia (HR: 1.25; 95% CI 1.14 to 1.37; p<0.001). Replacing 10% of UPF weight in the diet with equivalent, but less processed foods, was estimated to be associated with a 19% lower risk of all-cause dementia (HR: 0.81; 95% CI 0.74 to 0.89; p<0.001)	A higher UPF intake was significantly associated with a higher risk of all-cause dementia. Replacing UPF with less processed alternatives was associated with a reduced risk of developing dementia	N/A	Age, sex, education level, ethnicity, Townsend deprivation index, smoking status, drinking status, physical activity, BMI, sleep duration, CVD, family history of dementia, total energy intake, healthy diet score
Weinstein *et al*^25^	Cognitive decline	14 cognitive tests that were grouped into four domains. (1) Episodic memory: word list memory, word list recall and word list recognition (CERAD). (2) Attention/working memory: the diamond cancellation and the digit span (forward and backward) tests from the Wechsler Memory Scale-Revised (WMS-R). (3) Semantic categorisation: similarities, letter fluency and animal fluency tests. (4) Executive function: trail making test (A and B), CERAD-constructional praxis and digit symbol from the Wechsler Adult Intelligence Scale (WAIS)-Revised. Raw scores converted to Z scores	Mixed-effect models with random intercepts and slopes	Total UPF consumption was not associated with cognitive change during the follow-up (of any domain). Higher intake of UPF meat (executive function: β=−0.041, SE: 0.013; p=0.002 and global cognition: β=−0.026, SE: 0.010; p=0.011) and oils/spreads was associated with greater decline in executive function and global cognition (oils and spreads executive function: β=−0.037, SE: 0.014; p=0.006 and global cognition: β=−0.028, SE: 0.010; p=0.009)	In a type 2 diabetic cohort, total UPF intake was not associated with cognitive change. Particular subgroups of UPF may be harmful for cognitive health as ultra-processed meat and oils/spreads were associated with accelerated decline in executive functioning and global cognition	UPF subgroups	Age, sex, education, current smoking and physical activity, BMI, HbA1C, duration of diabetes, total cholesterol and blood pressure
Gomes Goncalves *et al*^26^	Cognitive decline	Six cognitive tests that were grouped into three domain composites. (1) Executive function: phonemic and semantic verbal fluency tests, and Trail-Making Test B. (2) Memory: immediate and delayed word recall, word recognition (CERAD). (3) Global: all cognitive tests. Raw scores converted to Z scores	Linear mixed-effects models with random intercepts and slopes	Individuals with UPF intake above the lowest quartile of consumption exhibited a 28% (β=−0.004; 95% CI −0.006 to −0.001; p=0.003) faster rate of global cognitive decline and a 25% (β=−0.003, 95% CI −0.005 to 0.000; p=0.01) faster rate of executive function decline compared with individuals in the lowest quartile of consumption. Age was an effect modifier in the association of UPF intake and cognitive function (p for interaction <0.001). Adhesion to a healthy diet was an effect modifier on the association between UPF and global cognitive function (p for interaction =0.04)	Higher consumption of UPFs was associated with a higher rate of global and executive function decline. Age and overall healthy eating patterns might modify the effect of UPF consumption on cognitive health	Age and diet quality	Age, sex, monthly income per capita in US dollars, education, race and ethnicity, BMI, diabetes, hypertension, CVD, depression, physical activity, smoking, alcohol consumption, total energy intake and adhesion to a healthy diet
Bhave *et al*^23^	Cognitive impairment and cognitive decline	Impairment was defined using performance relative to normative sample on memory (Word List Learning and Delayed Recall) and fluency assessments (Animal Fluency and Letter F Fluency). Incident cognitive impairment was defined as impairment on ≥1 memory assessment and ≥1 fluency assessment	Cox proportional hazards models	A 10% increase in UPF intake was associated with a 16% higher risk of cognitive impairment (HR; 1.16; 95% CI 1.09 to 1.24, p<0.001). Greater UPF intake was associated with increased cognitive decline in word learning (β=−0.0006; 95% CI−0.0009 to −0.0004; p<0.0001), delayed recall (β=−0.0005; 95% CI −0.0007 to −0.0003; p<0.0001), animal fluency (β=−0.0005; 95% CI −0.0007 to −0.0003; p<0.0001) and letter fluency (β=−0.0004; 95% CI −0.0007 to −0.0002; p<0.0001)	Higher UPF intake was associated with an increased risk of developing cognitive impairment. Greater UPF consumption was associated with accelerated cognitive decline		Age, race, sex, income, education, physical activity, BMI, hypertension, diabetes, CVD, smoking status, alcohol consumption, depressive symptoms, total energy intake, healthy diet score

BMI, body mass index; CVD, cardiovascular disorder; UPF, ultra-processed food.

## Data Availability

Data are available in a public, open access repository.

## What Is Already Known On This Topic

⇒ Ultra-processed food (UPF) intake has been linked to an increased risk of developing many adverse health outcomes.
⇒ Studies have begun investigating the cognitive effects of consuming UPFs, with conflicting results.

### What This Study Adds

⇒ This study provides an overview of the evidence for the associations between UPF intake, as defined by the NOVA classification system, and cognitive health outcomes.
⇒ Deleterious effects of UPF consumption on multiple cognitive health outcomes were identified, but the results suggest possible specificity in the relationship between UPF and brain health in terms of participant characteristics and/or subtype of UPF consumed.

### How This Study Might Affect Research, Practice Or Policy

⇒ Findings from this study might encourage future investigations of NOVA classified food intake and cognitive outcomes, with particular focus on exploring the possible moderating role of healthy dietary patterns and the heterogeneity across UPF type and participant health.
