# Supplementary material for: Ultra-processed food exposure and cognitive outcomes: a systematic review of observational studies
Source: BMJ Nutr Prev Health. Author manuscript; Available in PMC 2026 Oct 30. (PMC7619653; doi:10.1136/bmjnph-2025-001325)
Supplement: Supplementary file 1 [file EMS218752-supplement-Supplementary_file.docx]

**Supplementary Materials: Ultra-processed food exposure and cognitive outcomes: A systematic review of observational studies.**

1. Inclusion and exclusion criteria used in the selection of papers identified in the systematic search, to be included in this review.
2. Full search strategies by database
3. Quality of evidence of included studies reported via the Newcastle Ottawa Quality Assessment Scale for longitudinal studies.
4. Quality of evidence of included studies reported via the Newcastle Ottawa Quality Assessment Scale for cross-sectional studies.
5. Full text exclusions with reasons
6. PRISMA 2020 Checklist

Supplementary table 1: Inclusion and exclusion criteria used in the selection of papers identified in the systematic search, to be included in this review.

Inclusion criteria:

| Item | Definition |
| --- | --- |
| Study design | Original research studies published in a peer reviewed journal. Studies to be of observational design (cross-sectional, prospective cohort, case-control). |
| Exposure | Had to assess UPF intake, defined using the NOVA food classification system (must make a direct reference to UPFs and NOVA).  The study must measure overall dietary intake of UPFs (not measure the intake of a single UPF item). |
| Outcome | Must assess the association of UPF with a cognitive outcome (dementia, (mild) cognitive impairment, cognitive performance (of any cognitive domain), cognitive performance trajectories).  Dementia includes AD, VD, frontotemporal dementia or Lewy Body dementia (does not include early onset dementia). The definition of dementia had to be based on diagnostic criteria or data/hospital linkage. |
| Effect Size | Odds Ratio; Relative Risk; Hazards Ratio / beta coefficients (when assessing cognitive performance). |
| Participants | Human adult participants (over 18 years old) |

Exclusion criteria:

| Item | Definition |
| --- | --- |
| Study design | Animal studies. Reviews, case-reports, case series, conference abstracts, book chapters, editorials or letters, non- English language. |
| Exposure | Studies which measured the intake of only one type of UPFs. Studies that determine the processing level of foods using any other method than the NOVA classification system. Studies which assessed dietary patterns which (post hoc) are high in UPFs but did not directly assessing the relationship between UPFs and (cognitive) outcome. |
| Outcome | Non-dementia neurological disorders (e.g. stroke, Parkinsons, epilepsy). |
| Participants | Human participants under 18 years old. Non-human participants. |

Supplementary Table 2 : Full search strategies by database.

| Database | Search terms | Number articles returned |
| --- | --- | --- |
| MEDLINE, APA Psych Articles, PsycINFO and Embase through OVID | #1. ultra-processed food.ab. or ultra-processed food.ti. or ultraprocessed food.ab. or ultraprocessed food.ti. or ultra processed food.ab. or ultra processed food.ti. or UPF.ab. or UPF.ti. or NOVA.ab. or NOVA.ti.    #2. Dementia.ab. or Dementia.ti. or Alzheimer.ab. or Alzheimer.ti. or Lewy body.ab. or Lewy body.ti. or LBD.ab. or LBD.ti. or frontotemporal.ab. or frontotemporal.ti. or cognitive.ab. or cognitive.ti. or cognition.ab. or cognition.ti. or MCI.ab. or MCI.ti. or memory.ab. or memory.ti. or brain function.ab. or brain function.ti. or executive function.ab. or executive function.ti.    #3. 1 AND 2 | 22157          2918863      509 |
| PUBMED | #1. "Dementia"[Title/Abstract] OR "alzheimer"[Title/Abstract] OR "lewy body"[Title/Abstract] OR "LBD"[Title/Abstract] OR "frontotemporal"[Title/Abstract] OR "cognitive"[Title/Abstract] OR "cognition"[Title/Abstract] OR "MCI"[Title/Abstract] OR "memory"[Title/Abstract] OR "brain function"[Title/Abstract] OR "executive function"[Title/Abstract]    #2. "ultra processed food"[Title/Abstract] OR "ultra-processed food"[Title/Abstract] OR "ultraprocessed food"[Title/Abstract] OR "UPF"[Title/Abstract] OR "NOVA"[Title/Abstract]    3#. 1 AND 2 | 1,024,487          8,717        129 |

Supplementary Table 3: Quality of evidence of included studies reported via the Newcastle Ottawa Quality Assessment Scale for longitudinal studies.

| **Author, year** | Li et al., 2022 | Weinstien et al., 2023 | Gonçalves et al., 2023 | Bhave et al., 2024 |
| --- | --- | --- | --- | --- |
| **NOS component** |  |  |  |  |
| Representativeness of exposed cohort | a | a | a | a |
| Selection of non-exposed cohort | a | a | a | a |
| Ascertainment of exposure | c | c | c | c |
| Outcome of interest not present at start of study | a | a | a | a |
| Comparability of cohorts | a & b | a & b | a & b | a & b |
| Assessment of outcome | a | a | a | a |
| Follow up length | a | a | a | a |
| Follow up rate | b | b | b | c |
| **Total Score** | **8** | **8** | **8** | **7** |

Supplementary Table 4: Quality of evidence of included studies reported via the Newcastle Ottawa Quality Assessment Scale for cross-sectional studies.

| **Authors, Year** | **Representativeness of sample** | **Sample size** | **Non-respondents** | **Ascertainment of exposure** | **Confounding factors controlled.** | **Ascertainment of outcome** | **Statistics** | **Total score** |
| --- | --- | --- | --- | --- | --- | --- | --- | --- |
| Cardoso et al., 2022 | 1 | 0 | 0 | 0 | 2 | 2 | 1 | **6** |

Supplementary Table 5: Full text exclusions with reasons

| Authors, Year | Title | Exclusion reason |
| --- | --- | --- |
| Claudino et al., 2023 | Consumption of ultra-processed foods and risk for Alzheimer’s disease: a systematic review | Wrong publication type |
| Henney et al., 2024 | High intake of ultra-processed food is associated with dementia in adults: a systematic review and meta-analysis of observational studies | Wrong publication type |
| Pourmotabb et al., 2025 | The association of ultra-processed food intake with neurodegenerative disorders: a systematic review and dose-response meta-analysis of large-scale cohorts | Wrong publication type |
| Puig-Vallverd et al., 2022 | The association between maternal ultra-processed food consumption during pregnancy and child neuropsychological development: a population-based birth cohort study | Wrong population |
| Liu et al., 2023 | Association of ultraprocessed foods consumption and cognitive function among children aged 4-7 years: a cross-sectional data analysis | Wrong population |
| Wang et al., 2023 | Ultra-processed food consumption and risk of dementia and Alzheimer’s disease: Long-term results from the Framingham Offspring Study | Retracted article |
| Lourenco et al., 2024 | Quality of nutritional uptake of community-dwelling, care-dependent older adults in Zurich | Wrong outcome |
| Meeusen et al., 2024 | A decrease in ultra processed food intake is associated with improvements in metabolic parameters during a combined lifestyle intervention | Wrong outcome |
| Noll et al., 2022 | Life habits of postmenopausal women: Association of menopause symptom intensity and food consumption by degree of food processing | Wrong outcome |
| Flanagan et al., 2024 | Ultra-processed Food Intake is Associated with Cognitive Decline Among Older Puerto Rican Adults Living in Boston | Wrong publication type |
| Bhave et al., 2024 | Ultra-Processed Food Intake Predicts Stroke Risk in the Reasons for Geographic and Racial Differences in Stroke (REGARDS) study | Wrong outcome |
| Reis et al., 2023 | The Association Between Dementia and Nutritional Status in Elderly | Wrong population |

References

Claudino PA, Bueno NB, Piloneto S, Halaiko D, Azevedo de Sousa LP, Barroso Jara Maia CH, Netto BD. Consumption of ultra-processed foods and risk for Alzheimer’s disease: a systematic review. Frontiers in nutrition. 2024 Jan 15;10:1288749.

Henney AE, Gillespie CS, Alam U, Hydes TJ, Mackay CE, Cuthbertson DJ. High intake of ultra-processed food is associated with dementia in adults: a systematic review and meta-analysis of observational studies. Journal of neurology. 2024 Jan;271(1):198-210.

Pourmotabbed A, Talebi S, Mehrabani S, Babaei A, Khosroshahi RA, Bagheri R, Wong A, Ghoreishy SM, Amirian P, Zarpoosh M, Hojjati Kermani MA. The association of ultra-processed food intake with neurodegenerative disorders: a systematic review and dose-response meta-analysis of large-scale cohorts. Nutritional Neuroscience. 2025 Jan 2;28(1):73-86.

Puig-Vallverdú J, Romaguera D, Fernández-Barrés S, Gignac F, Ibarluzea J, Santa-Maria L, Llop S, Gonzalez S, Vioque J, Riano-Galan I, Fernández-Tardón G. The association between maternal ultra-processed food consumption during pregnancy and child neuropsychological development: A population-based birth cohort study. Clinical Nutrition. 2022 Oct 1;41(10):2275-83.

Liu S, Mo C, Lei L, Lv F, Li J, Xu X, Lu P, Wei G, Huang X, Zeng X, Qiu X. Association of ultraprocessed foods consumption and cognitive function among children aged 4–7 years: A cross-sectional data analysis. Frontiers in nutrition. 2023 Oct 10;10:1272126.

Wang K, Tang W, Hao X, Zhao J. Retracted: Ultra‐processed food consumption and risk of dementia and Alzheimer's disease: Long‐term results from the Framingham Offspring Study.

Lourenço PD, Angst L, Srinivasan M. Quality of nutritional uptake of community-dwelling, care-dependent older adults in Zurich. Journal of Dentistry. 2024 Sep 1;148:105155.

Meeusen R.E., Geurts K., Lissenberg W., Berk K., Van Rossum E. A decrease in ultra processed food intake is associated with improvements in metabolic parameters during a combined lifestyle intervention. Obesity Facts. 2024 Jan 17;10:209-2010

Noll M, Zangirolami-Raimundo J, Baracat EC, da Costa Louzada ML, Júnior JM, Sorpreso IC. Life habits of postmenopausal women: Association of menopause symptom intensity and food consumption by degree of food processing. Maturitas. 2022 Feb 1;156:1-1.

Flanagan K, Noel S, Tucker K, Zhang X. Ultra-Processed Food Intake Is Associated With Cognitive Decline Among Older Puerto Rican Adults Living in Boston. Current Developments in Nutrition. 2024 Jul 1;8.

Bhave VM, Oladele C, Ament Z, Kijpaisalratana N, Jones A, Couch C, Patki A, Garcia Guarniz AL, Irvin MR, Kimberly WT. Ultra-Processed Food Intake Predicts Stroke Risk in the Reasons for Geographic and Racial Differences in Stroke (REGARDS) Study. Stroke. 2024 Feb;55:A63

Reis M, Cebola M. The Association Between Dementia And Nutritional Status In Elderly. Clinical Nutrition ESPEN. 2023 Apr 1;54:688.

Supplementary 6: PRISMA 2020 Checklist

| **Section and Topic** | **Item #** | **Checklist item** | **Location where item is reported** |
| --- | --- | --- | --- |
| **TITLE** | | |  |
| Title | 1 | Identify the report as a systematic review. | Y |
| **ABSTRACT** | | |  |
| Abstract | 2 | See the PRISMA 2020 for Abstracts checklist. | Y |
| **INTRODUCTION** | | |  |
| Rationale | 3 | Describe the rationale for the review in the context of existing knowledge. | Y |
| Objectives | 4 | Provide an explicit statement of the objective(s) or question(s) the review addresses. |  |
| **METHODS** | | |  |
| Eligibility criteria | 5 | Specify the inclusion and exclusion criteria for the review and how studies were grouped for the syntheses. | Y |
| Information sources | 6 | Specify all databases, registers, websites, organisations, reference lists and other sources searched or consulted to identify studies. Specify the date when each source was last searched or consulted. | Y |
| Search strategy | 7 | Present the full search strategies for all databases, registers and websites, including any filters and limits used. | Y |
| Selection process | 8 | Specify the methods used to decide whether a study met the inclusion criteria of the review, including how many reviewers screened each record and each report retrieved, whether they worked independently, and if applicable, details of automation tools used in the process. | Y |
| Data collection process | 9 | Specify the methods used to collect data from reports, including how many reviewers collected data from each report, whether they worked independently, any processes for obtaining or confirming data from study investigators, and if applicable, details of automation tools used in the process. | Y |
| Data items | 10a | List and define all outcomes for which data were sought. Specify whether all results that were compatible with each outcome domain in each study were sought (e.g. for all measures, time points, analyses), and if not, the methods used to decide which results to collect. | Y |
|  | 10b | List and define all other variables for which data were sought (e.g. participant and intervention characteristics, funding sources). Describe any assumptions made about any missing or unclear information. | Y |
| Study risk of bias assessment | 11 | Specify the methods used to assess risk of bias in the included studies, including details of the tool(s) used, how many reviewers assessed each study and whether they worked independently, and if applicable, details of automation tools used in the process. | Y |
| Effect measures | 12 | Specify for each outcome the effect measure(s) (e.g. risk ratio, mean difference) used in the synthesis or presentation of results. | Y |
| Synthesis methods | 13a | Describe the processes used to decide which studies were eligible for each synthesis (e.g. tabulating the study intervention characteristics and comparing against the planned groups for each synthesis (item #5)). | Y |
|  | 13b | Describe any methods required to prepare the data for presentation or synthesis, such as handling of missing summary statistics, or data conversions. |  |
|  | 13c | Describe any methods used to tabulate or visually display results of individual studies and syntheses. |  |
|  | 13d | Describe any methods used to synthesize results and provide a rationale for the choice(s). If meta-analysis was performed, describe the model(s), method(s) to identify the presence and extent of statistical heterogeneity, and software package(s) used. | Y |
|  | 13e | Describe any methods used to explore possible causes of heterogeneity among study results (e.g. subgroup analysis, meta-regression). |  |
|  | 13f | Describe any sensitivity analyses conducted to assess robustness of the synthesized results. |  |
| Reporting bias assessment | 14 | Describe any methods used to assess risk of bias due to missing results in a synthesis (arising from reporting biases). |  |
| Certainty assessment | 15 | Describe any methods used to assess certainty (or confidence) in the body of evidence for an outcome. |  |
| **RESULTS** | | |  |
| Study selection | 16a | Describe the results of the search and selection process, from the number of records identified in the search to the number of studies included in the review, ideally using a flow diagram. | Y |
|  | 16b | Cite studies that might appear to meet the inclusion criteria, but which were excluded, and explain why they were excluded. |  |
| Study characteristics | 17 | Cite each included study and present its characteristics. | Y |
| Risk of bias in studies | 18 | Present assessments of risk of bias for each included study. | Y |
| Results of individual studies | 19 | For all outcomes, present, for each study: (a) summary statistics for each group (where appropriate) and (b) an effect estimate and its precision (e.g. confidence/credible interval), ideally using structured tables or plots. | Y |
| Results of syntheses | 20a | For each synthesis, briefly summarise the characteristics and risk of bias among contributing studies. | Y |
|  | 20b | Present results of all statistical syntheses conducted. If meta-analysis was done, present for each the summary estimate and its precision (e.g. confidence/credible interval) and measures of statistical heterogeneity. If comparing groups, describe the direction of the effect. |  |
|  | 20c | Present results of all investigations of possible causes of heterogeneity among study results. | Y |
|  | 20d | Present results of all sensitivity analyses conducted to assess the robustness of the synthesized results. |  |
| Reporting biases | 21 | Present assessments of risk of bias due to missing results (arising from reporting biases) for each synthesis assessed. |  |
| Certainty of evidence | 22 | Present assessments of certainty (or confidence) in the body of evidence for each outcome assessed. |  |
| **DISCUSSION** | | |  |
| Discussion | 23a | Provide a general interpretation of the results in the context of other evidence. | Y |
|  | 23b | Discuss any limitations of the evidence included in the review. | Y |
|  | 23c | Discuss any limitations of the review processes used. |  |
|  | 23d | Discuss implications of the results for practice, policy, and future research. | Y |
| **OTHER INFORMATION** | | |  |
| Registration and protocol | 24a | Provide registration information for the review, including register name and registration number, or state that the review was not registered. | Y |
|  | 24b | Indicate where the review protocol can be accessed, or state that a protocol was not prepared. | Y |
|  | 24c | Describe and explain any amendments to information provided at registration or in the protocol. | Y |
| Support | 25 | Describe sources of financial or non-financial support for the review, and the role of the funders or sponsors in the review. | Y |
| Competing interests | 26 | Declare any competing interests of review authors. | Y |
| Availability of data, code and other materials | 27 | Report which of the following are publicly available and where they can be found: template data collection forms; data extracted from included studies; data used for all analyses; analytic code; any other materials used in the review. | Y |
